# Assessment of Health-Related Quality of Life and Its Determinants in Type 2 Diabetes Mellitus Patients: A Cross-Sectional Study

**DOI:** 10.7759/cureus.66055

**Published:** 2024-08-03

**Authors:** Amit Kumar Mishra, Manoj Kumar Choudhary, Chandan Kumar, Anand Kishor, Anjali Kumari

**Affiliations:** 1 Department of General Medicine, Indira Gandhi Institute of Medical Sciences, Patna, IND

**Keywords:** hrqol, euroqol-visual analogue scale, eq-5d-5l questionnaire, quality of life, coronary artery disease, hyperglycemia, hba1c, type 2 diabetes

## Abstract

Background

Type 2 diabetes mellitus is a complex metabolic disorder associated with several complications that determine the quality of life of the patients. Health-related quality of life (HRQoL) is a measurable outcome of the self-perception of a patient’s health which is affected due to age, lifestyle changes, medication, and treatment modalities. This study was undertaken to understand the impact of individual parameters of age, medication type and duration, diabetes-associated complications, and levels of glycated hemoglobin (HbA1c) on the quality of life (QoL) of the patient.

Methodology

This single-center prospective, cross-sectional study was conducted at the Indira Gandhi Institute of Medical Sciences (IGIMS), Patna, Bihar, India. Participants were recruited from the Outpatient Department of General Medicine, IGIMS. HRQoL was measured using a validated and reliable EuroQol 5-dimensions 5-levels (EQ-5D-5L) questionnaire developed by the EuroQol Research Foundation, along with the EuroQol-Visual Analogue Scale (EQ-VAS). The eligibility criteria included adult diabetic patients above 18 years of age with complete medical records, who had been treated at the outpatient department for a minimum of three months and could be interviewed.

Results

The results from this study show that 46% of the patients belonged to the age group of 45-65 years. The quality of health index scores and EQ-VAS scores significantly correlated with age (p-values: 1.11 e^-4 ^and 3.09 e^-5^; <0.05). Of the subjects, 66.4%, 6.7%, and 26.8% were under oral hypoglycaemic agents (OHA), insulin, and both insulin with OHA medications respectively. HbA1C levels were statistically significantly correlated with mobility, self-care, usual activities, pain or discomfort, and anxiety or depression (p-value 0.032; <0.05), along with self-perception of the patient’s health (p-value 0.00026; <0.05). Also, the perception of having slight problems in mobility, self-care, usual activities, pain or discomfort, and anxiety or depression was similar irrespective of gender (EQ-5D-5L score for males: 9.47 and females: 9.3). Despite suffering from diabetes-associated chronic complications, 60.5% of the subjects perceived their overall health to be good as indicated by the scores.

Conclusion

The self-perception of HRQoL concerning mobility, self-care, usual activities, pain or discomfort, and anxiety or depression was correlated with age, duration of anti-diabetic medication, and HbA1C level. Good mobility, self-care, and performing usual activities reduce anxiety or depression as opposed to age, pain, and discomfort. However, the subjects in this study cohort perceived overall good health in themselves in terms of EQ-VAS and 5D-5L scores, indicating effective diabetic care and management options available to them.

## Introduction

Diabetes mellitus is a metabolic disorder marked by high blood glucose levels due to gradual but progressive deterioration of the pancreatic beta-cell function. With time, a decrease in insulin levels and insulin resistance leads to chronic hyperglycemia. There are three types of hyperglycemia: type 1, type 2, and gestational diabetes. More than 95% of people with diabetes suffer from type 2 diabetes. As per the WHO statistics, between 2000 and 2019, there was a 3% increase in age-standardized mortality rates from diabetes. Also, another 4,60,000 kidney-related deaths were due to diabetes, and high blood glucose caused around 20% of cardiovascular deaths [1].

Diabetic patients are often exposed to a higher risk of micro- and macro-vascular complications leading to nephropathy, neuropathy, retinopathy, and coronary artery disease (CAD), thereby impacting the quality of life of the individual. The aftermath of such a situation is higher healthcare expenditure and deteriorating socioeconomics. Nephropathy arises due to chronic renal insufficiency, inflammation of kidneys, glomerular damage, and both intrarenal and extrarenal atherosclerosis. Negative impacts on both autonomic and peripheral nervous systems and sensory nerve endings of limbs, primarily the lower limbs, lead to loss of sensation in these areas and hence, neuropathy [2]. Another major complication is diabetic retinopathy which manifests itself due to the dysfunction of epithelial cells and pericytes of the retina. The microvasculature of the retina is also affected by the advanced glycation products and oxidative stress. Endothelial dysfunction and atherosclerosis are the main contributors to hyperglycaemic CAD. The outcomes of these long-term complications finally culminate in premature death [3].

Psychological attributes of diabetes influence self-care behavior, glycemic control, and thus quality of life (QoL) [4]. The WHO states QoL as “an individual’s perception of their position in life in the context of the culture and value systems in which they live and about their goals, expectations, standards, and concerns.” According to the Centers for Disease Control and Prevention (CDC), health-related QoL (HRQoL) is an individual’s or group’s self-perceptive physical and mental health surveillance over time [5]. It is the personal perception of the patient, pointing to the comfort areas that are influenced by his/her health status. It contains patient-oriented and patient-reported outcome measures that evaluate his/her physical function and psychological status. The psychological symptoms of hyperglycemic stress such as denial, anger, anxiety, depression, desolation, isolation, shock, guilt, and frustration make the self-management of blood sugar more difficult, which affects the patient’s HRQoL. Hence, there is an urgent need to gauge a patient’s social, physical, and mental well-being to improve upon their self-care attitude and carve out disease management and lifestyle strategies. This will also lead to compliance with the ongoing therapy thereby avoiding further deterioration of their well-being. In a systematic review of HRQoL, physical function and movement ability were found to be important indicators [6]. The multifactorial relationship between diabetes and associated psychosocial disorders can impact self-care behavior, glycemic control, and hence QoL [7].

Among other available options, the standardized EuroQol 5-dimensions 5-levels (EQ-5D-5L) questionnaire has attracted attention in recent times due to its patient preference-based multiattribute utility [8]. In this study, we employed the EQ-5D-5L questionnaire to gain insight into the patient’s perspective in combating the disease. The objective of this study was to understand the impact of the individual parameters of age, diabetes duration, medication type and duration, diabetes-associated complications, and levels of glycated hemoglobin (HbA1c) on HRQoL scores and, hence, the QoL of the patients. This study will aid in fine-tuning the ways to assess and devise effective glycemic management strategies for hyperglycemia and its associated complications through an HRQoL analysis.

## Materials and methods

This was a single-center, prospective, cross-sectional study, conducted at Indira Gandhi Institute of Medical Sciences (IGIMS), Patna, Bihar, India. The study was approved by the Institutional Ethics Committee, IGIMS (approval number: 1205/IEC/IGIMS/2023). The participants were recruited from the Outpatient Department of General Medicine, IGIMS.

Study participants

Inclusion criteria were adult patients (aged ≥ 18 years) who had been diagnosed with diabetes and been on treatment for at least three months at the outpatient department, who could be interviewed, and who had complete medical records of laboratory tests and clinical parameters. Individuals <18 years of age are not mature enough to deal with pain, anxiety, and depression, and to carry out self-care activities, and hence were excluded from the study. Since the study is based on a questionnaire addressed to the patients themselves, it was comprehended that the individuals who were unable to communicate due to severe illnesses, psychiatric disorders, or neurological illnesses would not be in a condition to answer the questions correctly thereby leading to misleading and biased results, and were thus excluded. Diabetic pregnant mothers might already have difficulty in carrying out usual activities, mobility, anxiety, and discomfort issues which could have influenced the results, and were also excluded. Patients with incomplete medical records and/or who refused to participate were also excluded from this study. 

The sample size of 119 participants in this study was based on the number of individual participants who met the inclusion and exclusion criteria during the study period. Kaiser-Meyer-Olkin test (KMO≥0.8) was used to find the adequacy of sample size for factor analysis with Bartlett's test p≤0.05.

Tools

HRQoL was measured using a validated and reliable tool developed by the EuroQol Research Foundation, the EQ-5D-5L questionnaire [8]. Permission to use the questionnaire for this study has been taken from the EuroQol Research Foundation. This test had sufficient reliability and validity after translation [8-10]. The EQ-5D-5L has two sections. The first section involves patients’ self-testified components specifying their health status. It has five domains (mobility, self-care, usual activities, pain or discomfort, and anxiety or depression). Each domain has five difficulty levels: No problems, slight problems, moderate problems, severe problems, and extreme problems.

The questionnaire was set in English and then translated to the local language (Hindi) by experts to adhere to uniformity. The English version of the questionnaire is included in the Appendices.

The second section, the EQ-VAS (see Appendices), is an instrument used for the subjective assessment of the current health status from the patient’s self-perspective. It is a vertical visual analog scale with values between 100 (best imaginable health) and 0 (worst imaginable health). The patients evaluate their overall health status on this scale.

Data collection

Data were collected through a face-to-face interview, a review of laboratory records, and a structured questionnaire comprising items assessing the HRQoL of the participants using the EQ-5D-5L and EuroQol-Visual Analogue Scales (EQ-VAS). Participants were asked to choose a level that reflected their current state of health for each dimension. Then, their EQ-5D-5L utility scores were calculated, which is a level summary score (LSS) ranging from 5 to 25, with 5 being no problem on any dimension and 25 being extreme problems on all dimensions. It is calculated as for no problems-1, slight problems-2, moderate problems-3, severe problems-4 and extreme problems-5. So, the lower the EQ-5D-5L utility score, the better the HRQoL of the patients.

In the EQ-VAS, each patient self-rated their state of health.

The sociodemographic characteristics such as age, sex, and marital status and diabetes-related clinical parameters such as duration of diagnosis, medications, diabetes-related chronic complications, and glycated hemoglobin were also collected.

Statistical analysis

The data entered into a Microsoft Excel sheet (Microsoft Corporation, Redmond, Washington, United States) was transported to IBM SPSS Statistics for Windows, Version 25.0 (Released 2017; IBM Corp., Armonk, New York, United States) for analysis. Descriptive statistics such as mean and standard deviation (SD) were used to obtain results for continuous variables. Paired sample t-tests were used to calculate the p-values and hence examine the effects of various factors in HRQoL scores between groups. Linear regression analysis was used to identify the association between the independent variables and the HRQoL. Mean EQ-VAS was computed along with standard deviation. A p-value < 0.05 at a 95% confidence interval (CI) was considered to be statistically significant.

## Results

A total of 119 patients were included in the study. The Kaiser-Meyer-Olkin value obtained was 0.832 with a good Bartlett test of sphericity (p < 0.01), indicating appropriate sample adequacy. The gender distribution shows that among the participants, 76 (64%) were males and 43 (36%) were females. All the participants were married except one 40-year-old unmarried female (Table 1).

**Table 1:** Variable data of the subject population studied (N=119) OHA: oral hypoglycemic agents; EQ-5D-5L: EuroQol 5-Dimensions 5-Levels questionnaire; LSS: level summary score; EQ-VAS: EuroQol-Visual Analog Scale

Variables	Frquency	Percentage
Age		
30-40 years	12	10
40-50 years	33	27.7
50-60 years	28	23.5
60-70 years	34	28.6
70-80 years	10	8.4
80-90 years	2	1.7
Gender		
Male	76	64
Female	43	36
Marital Status		
Married	118	99.1
Unmarried	1	0.1
Medication		
OHA	79	66.4
Insulin	8	6.7
Insulin + OHA	32	26.8
Chronic complications		
None	76	63.8
Yes	43	36.2
EQ-5D-5L LSS scores		
4-6	28	23.5
6-8	32	26.8
8-10	12	10
10-12	7	5.9
12-14	14	11.7
14-16	8	6.7
16-18	8	6.7
18-20	6	7
20-22	4	3.3
EQ-VAS scores		
20-40	13	11
40-60	26	21.8
60-80	25	21
80-100	55	46.2

Age

**Figure 1:**
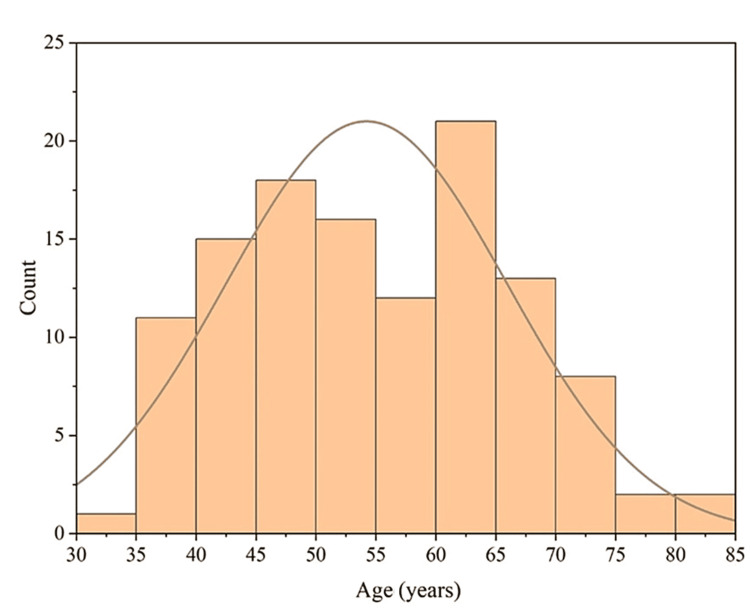
Histogram representing the age distribution of the study population (N=119)

The mean age of the population in this study was 55 years with the distribution of 56% of the diabetic subjects within the age range of 45-65 years. The graphical representation of the age of study participants followed a symmetrical, unimodal, normal distribution curve (Figure 1). The mean EQ-5D-5L for the HRQoL score obtained here was 10. The statistical analysis of age with HRQoL scores showed a significant correlation between them with a p-value of 1.11e^-4^(<0.5) and provided the basis for the rejection of the null hypothesis. The R-squared value (R^2^) of 0.47 showed that the data had incorporated 47% variability with moderate correlation. The EQ-VAS scores had a median value of 75 with a mean of 70. The p-value was found to be 213.09 e^-5^ (p-value <0.05) with R^2^ of 0.5 (data variability is 50%). Hence, both the EQ-5D-5L and EQ-VAS were significantly correlated with the age of the subject population.

Medication

**Figure 2:**
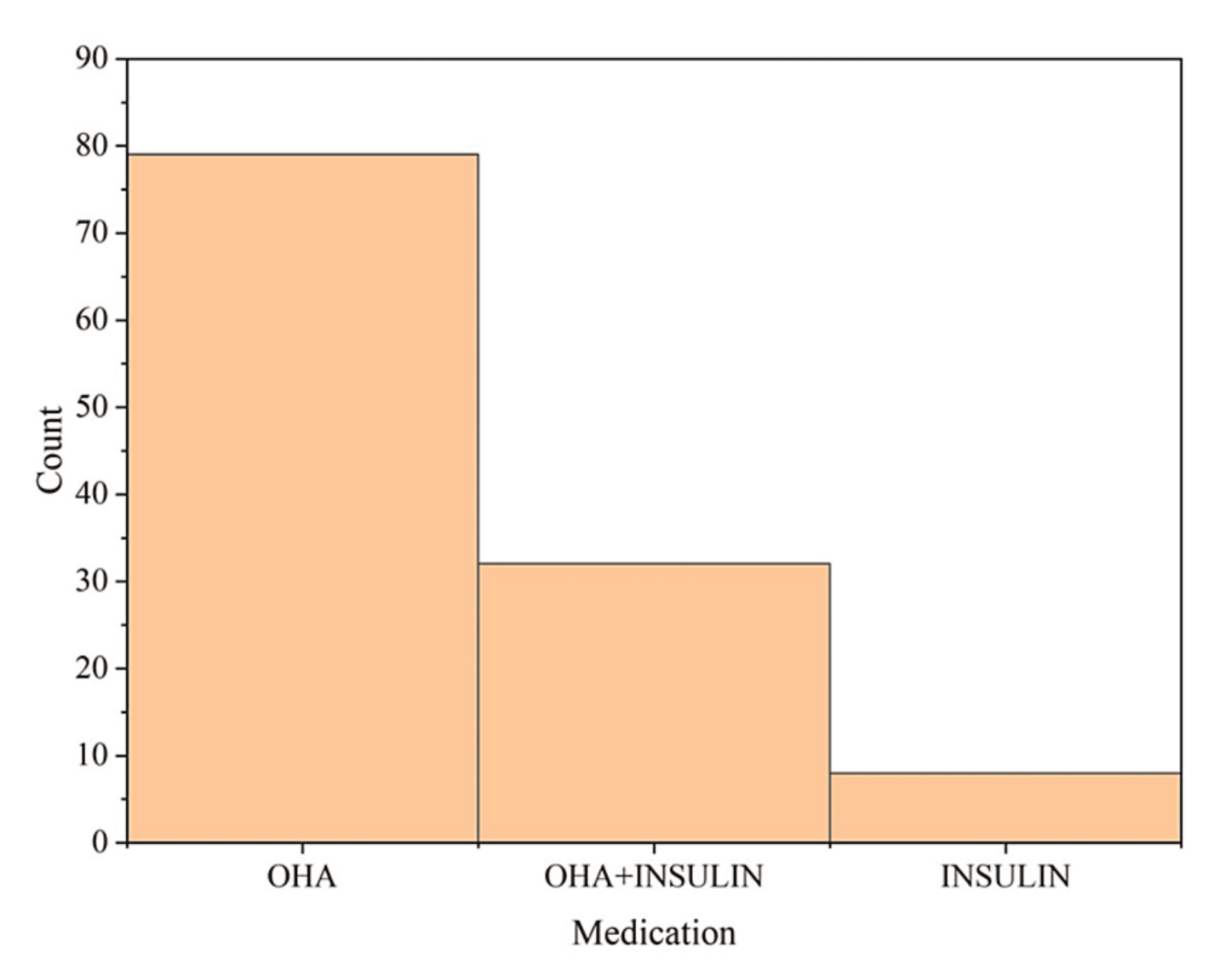
Distribution of the type of medication in the study population OHA: oral hypoglycaemic agents

All the subjects in this study were undergoing medication for hyperglycemia ranging from oral hypoglycaemic agents (OHA) (n=79; 66.4%), insulin (n=8; 6.7%), and OHA with insulin (n=32; 26.8%) (Figure 2). The statistics showed that though diabetes is a complex disease affecting the metabolism and many organs and their functioning, the management strategy was majorly governed by OHA in this study, reflecting a p-value <0.05.

Medication duration

**Figure 3:**
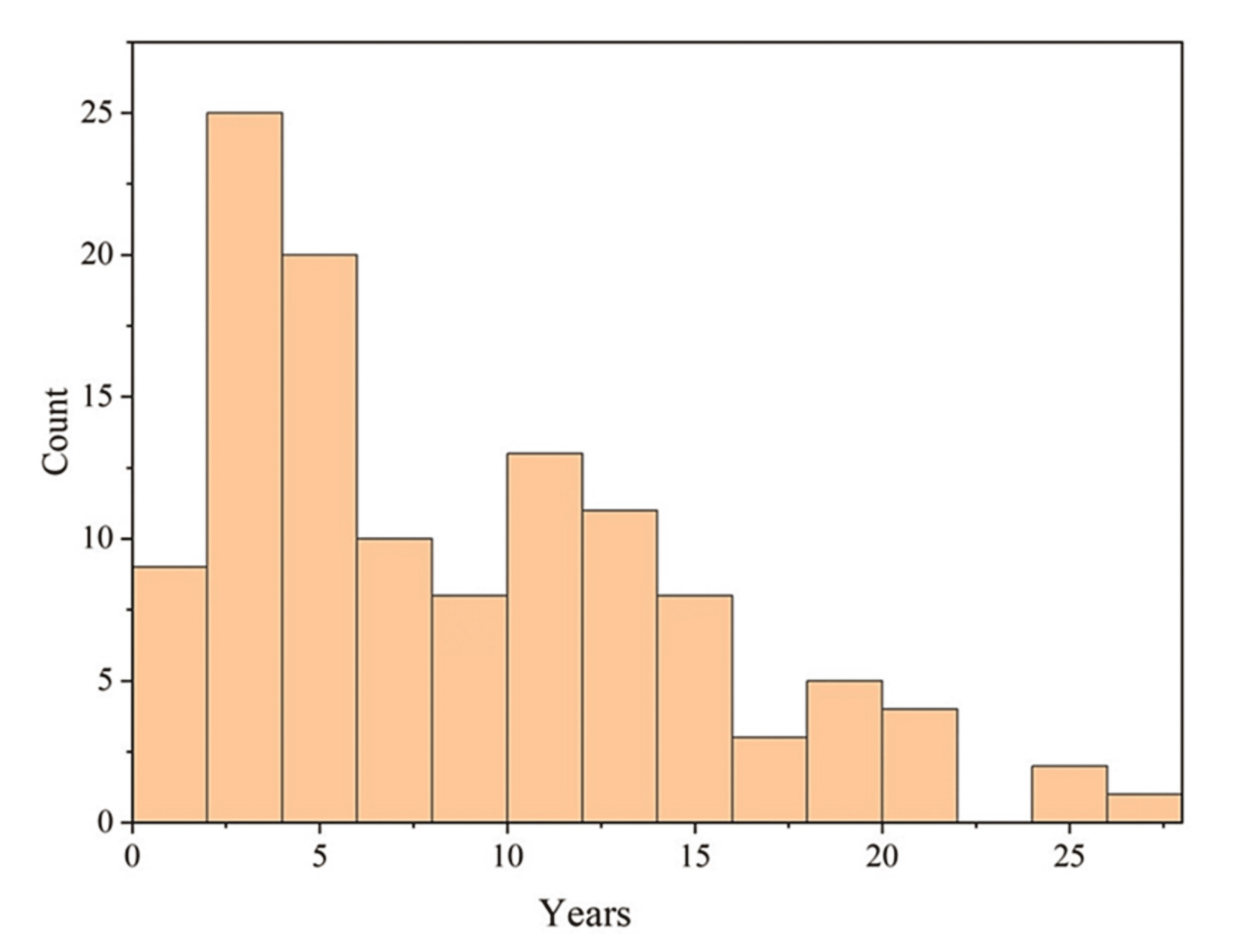
Distribution of duration of medication undergone by the study population

The patients were under diabetic medication for a duration spanning 1-25 years (Figure 3). The statistical correlation of medication duration with EQ-5D-5L score displayed p-value and the goodness of fit, R^2^ value of 2.66e^-7^(<0.05), and 0.6, respectively. Thus, the relationship was statistically significant. For EQ-VAS, the p-value was 6.1e^-3^ (<0.05) and R^2^ was 0.6, which again reflected the statistical significance of the parameters of medication duration.

HbA1c

**Figure 4:**
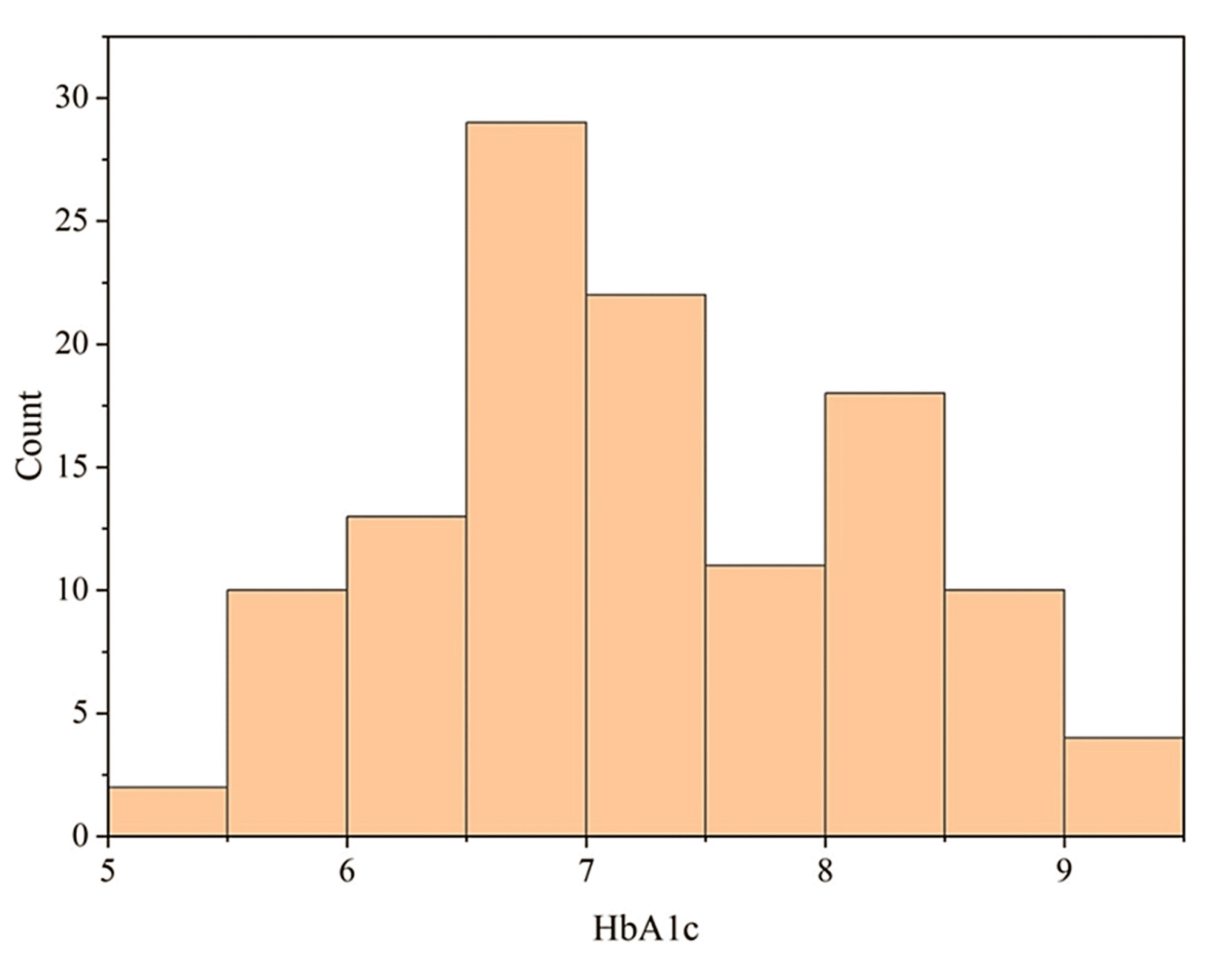
Distribution of HbA1C percentage among the study population (N=119) HbA1C: glycated hemoglobin

In this study, we wanted to identify whether there existed any relationship between HbA1c and QoL of the patients. Figure 4 represents the distribution of HbA1C percentage throughout the study cohort.

Our sample cohort had quite poor glycaemic control, as obvious from the HbA1C levels, with mean HbA1C being 7.24%. The International Diabetes Federation (IDF) recommends ≥6.5% (48 mmol/mol) as a hyperglycaemic or diabetic condition. Of the participants, 99 (79%) had HbA1C levels ≥6.5% during the course of the study. The summary output of regression analysis between the HbA1C with EQ-5D-5L and EQ-VAS scores reflects that there existed a significant relationship between HbA1C and EQ-VAS scores (p-value 0.00026; <0.05), and EQ-5D-5L (p-value = 0.032; <0.05). It may be inferred that HbA1C levels were significantly correlated with mobility, self-care, usual activities, pain or discomfort, and anxiety or depression of the patient, along with self-perception of his/her health.

Chronic complications

**Figure 5:**
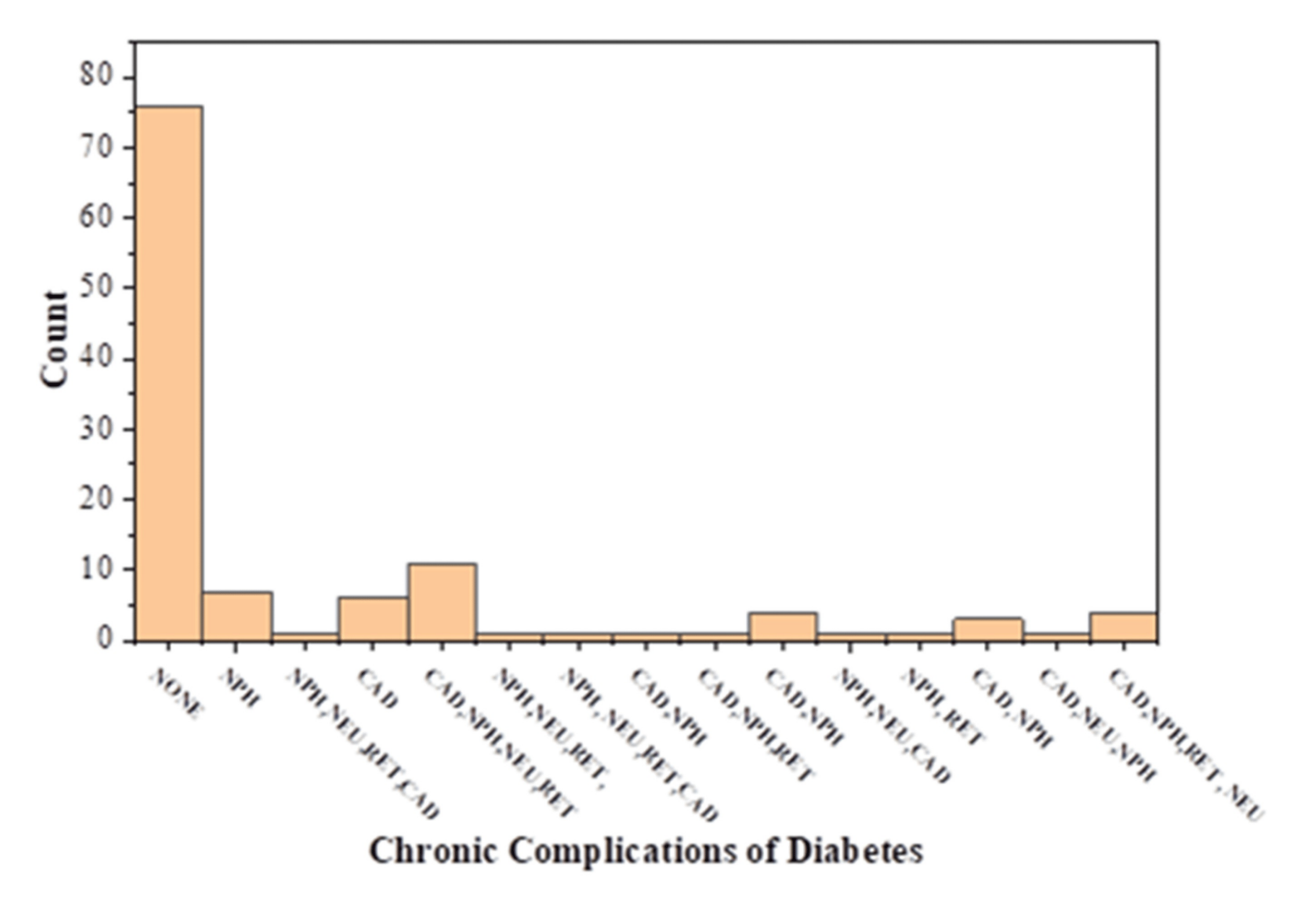
Frequency of diabetes-associated chronic complications among the study population (N=119) NPH: nephropathy; NEU: neuropathy; RET: retinopathy; CAD: coronary artery disease

**Figure 6:**
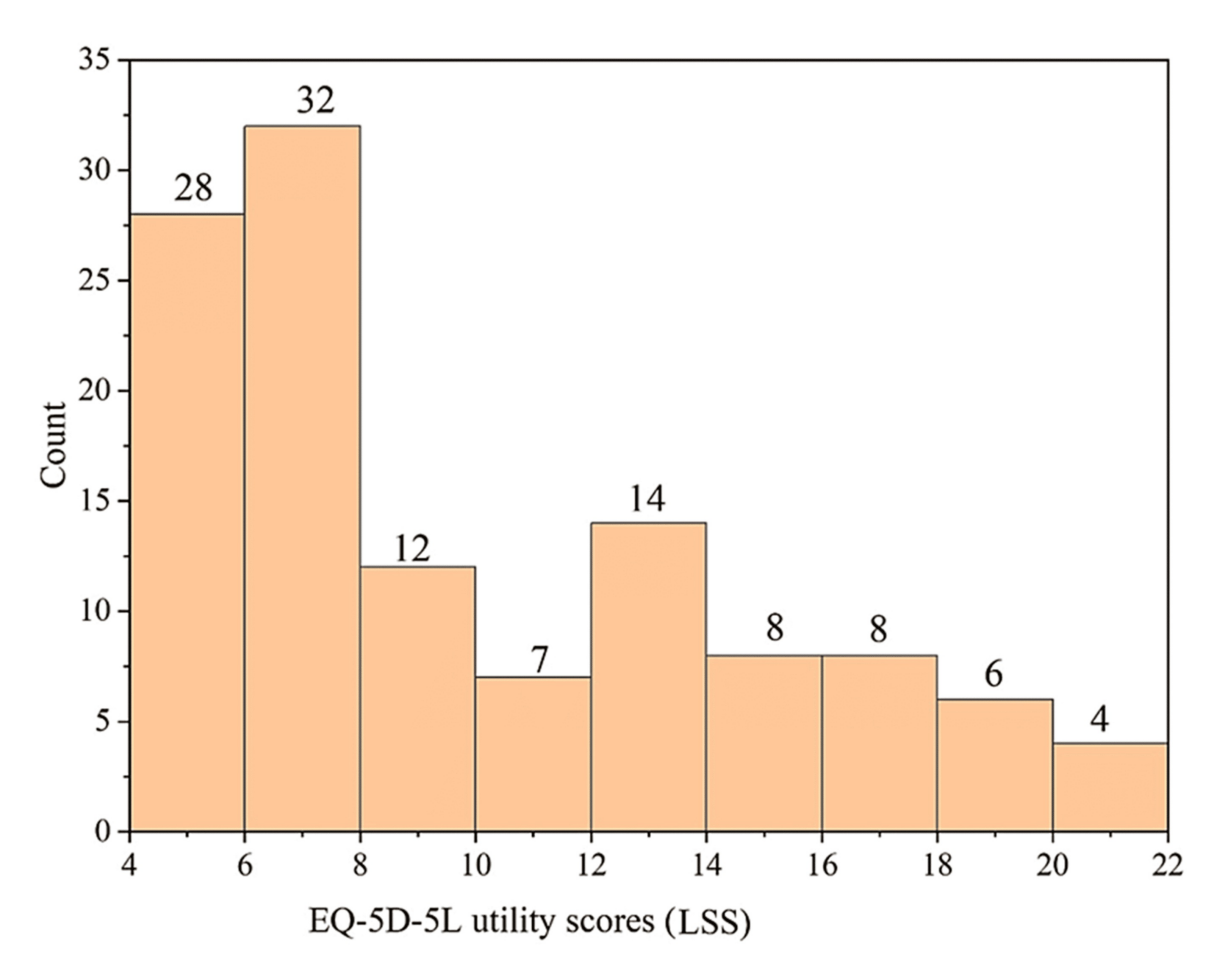
Distribution of EQ-5D-5L scores among the study population (N=119) EQ-5D-5L: EuroQol 5-Dimensions 5-Levels questionnaire; LSS: level summary score

**Figure 7:**
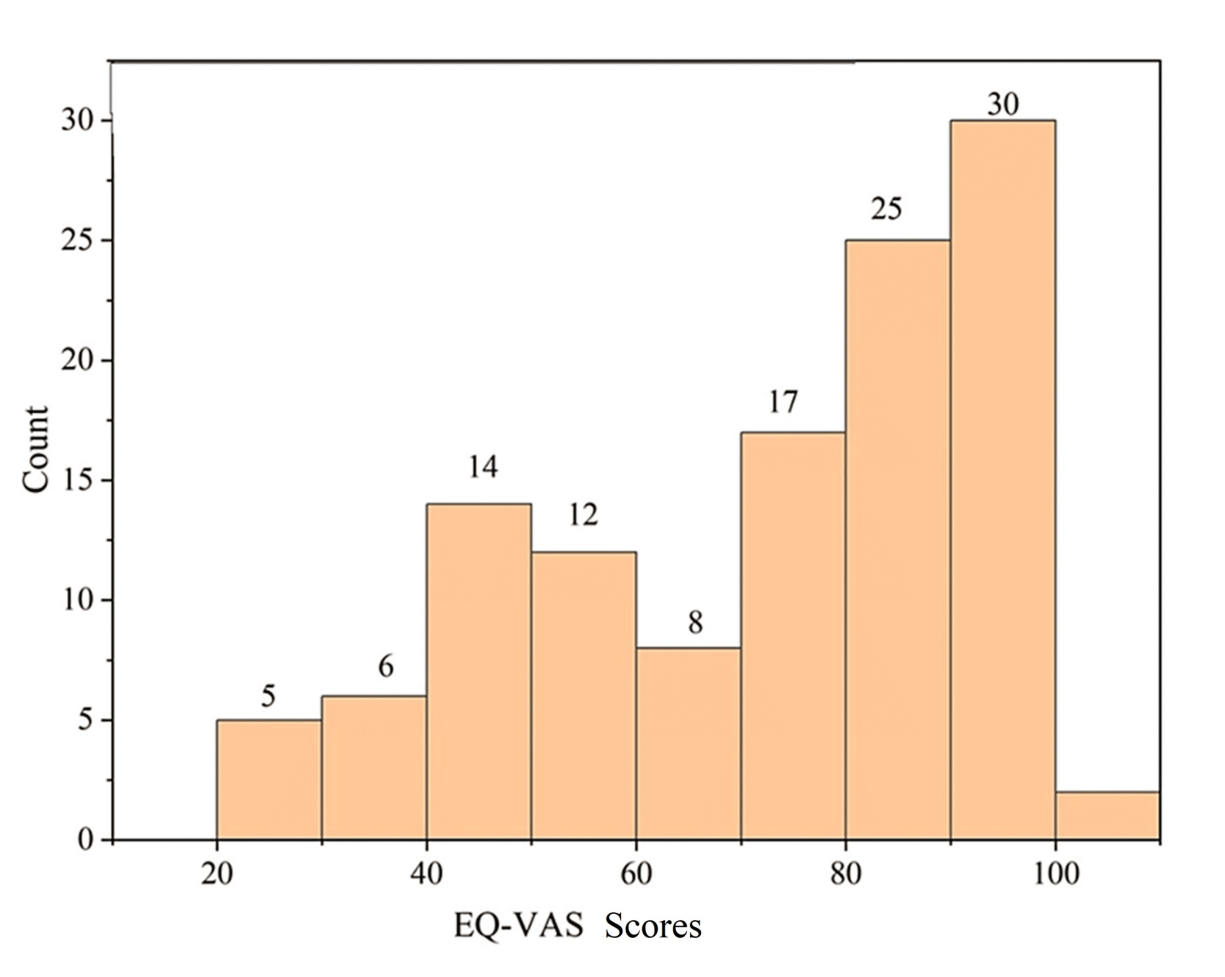
Distribution of EQ-VAS scores among the study population (N=119) EQ-VAS: EuroQol-Visual Analog Scale

Some of the participants in this study (n=43; 36%) suffered from chronic complications like nephropathy, neuropathy, retinopathy, and coronary artery disease (CAD) (Figure 5). Of these, 37 (31%), 17 (14%), 20 (17%), and 33 (32%) suffered from nephropathy, neuropathy, retinopathy, and CAD, respectively, either by itself or in combination with others. Though the participants perceived their overall health to be good according to the low EQ-5D-5L (Figure 6) and high EQ-VAS scores (Figure 7), the contributory factor was the absence of chronic complications.

EQ-5D-5L and EQ-VAS scores

The distribution of the individuals within a particular range of EQ-5D-5L and EQ-VAS scores is tabulated in Table 1. For 72 (60%) participants, the self-perceived and self-conceived EQ-5D-5L scores were 4-10 (Figure 6). Similarly, their overall health score on the EQ-VAS scale was within 70-100 (Figure 7). Both males and females had average EQ-5D-5L scores above 9 (males: 9.47 and females: 9.3).

**Figure 8:**
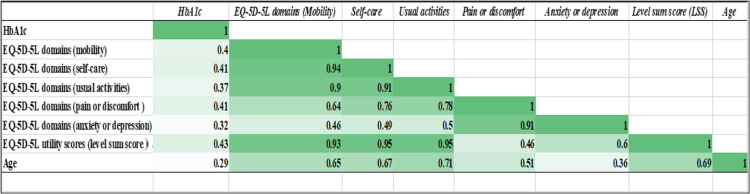
Heat map of correlation between HbA1C, EQ-5D-5L domains*, level sum scores, and age *mobility, self-care, usual activities, pain or discomfort, anxiety or depression HbA1C: glycated hemoglobin; EQ-5D-5L: EuroQol 5-Dimensions 5-Levels questionnaire

A correlation heatmap was generated to understand the relationship between HbA1C, EQ-5D-5L domains (mobility, self-care, usual activities, pain or discomfort, anxiety or depression), LSS, and age. The data in the correlation heat map were structured to represent the percentage of highly correlated events in dark green and the percentage of poorly correlated events in light green (Figure 8).

## Discussion

Early detection of diabetes is possible now due to easy access to clinical facilities and improved and standardized diagnostic methods. Since diabetes is a chronic metabolic disorder compelling lifelong medication and testing, it drastically affects the QoL until the proper management and medications are considered [11]. This study is a step towards understanding and fine-tuning the means to develop glycemic control strategies for both hyperglycemia and the associated complications through the HRQoL analyses. Demographic, medication, and physiological parameters along with self-perceptive QoL indices were considered (Table 1) for scoring from best health (100) to worst health (0).

The mean age of participants in this study is 55 years. Most studies show higher age (>50 years) to be vulnerable to poor QoL scores. From our study, we also were able to establish a statistically significant relationship between age and EQ-5D-5L and EQ-VAS scores because of quite low p-values (1.11e^-4^ and 3.09e^-5^; <0.05) [12-14]. Both genders had average EQ-5D-5L scores above 9 (males: 9.47 and females: 9.3). Hence, the perception of having slight problems in mobility, self-care, usual activities, pain or discomfort, and anxiety or depression was similar irrespective of the gender though males fared slightly better than females. Some studies found females to have poor predictors for QoL scores [14,15].

HbA1c is the golden yardstick for the diagnosis of diabetes. It correlates with the glucose measurement and is hence an objective measure of the glycaemic status of the patient [16]. The statistics of the diagnostic criterion of hyperglycemia, HbA1C level, demonstrate that 21% of the subjects had HbA1C <6.5%. However, quite prominently, 99 (79%) participants reflected hyperglycemia with HbA1C > 6.5%. OHAare orally administered drugs that are used to reduce blood sugar levels. OHA stimulate the pancreatic beta-cell function to produce and secrete insulin. Insulin is the peptide hormone released by pancreatic beta cells. These are generally used for effective management of type 2 diabetes mellitus. All the subjects in this study were undergoing medication for hyperglycemia ranging from OHA, insulin, and both OHA with insulin. Of the subjects, 79 (66.4%), eight (6.7%), and 32 (26.8%) were under OHA, insulin, and both medications (Figure 2) showing compliance to medication and a willingness to control hyperglycemia. A total of 53 (44.5%) patients were diagnosed with the disease within five years in contrast to three (2.5%) cases detected 20-30 years back. Thus, the risk of diabetes is more prominent within 10 years (>60%) [17,18].

The correlation heat map (Figure 8) offers insight into the relationship between various parameters. The level of correlation of HbA1C with all EQ-5D-5L parameters is less (0.32-0.43), and more so with age (0.29). If the subjects have good mobility, they can take care of themselves (0.94), perform usual activities (0.90), and have less anxiety or depression (0.46). However, more pain and discomfort lead to more anxiety and depression (0.91). Higher age leads to more pain (0.51) and anxiety or depression (0.36). LSS is also opposingly impacted by pain, discomfort (0.46), and anxiety and depression (0.60). The associated comorbidities of nephropathy neuropathy, retinopathy, and CAD not only affect the economics but also the QoL of the patients and their families. Of the chronically affected participants, 37 (31%), 17 (14%), 20 (17%), and 33 (32%) suffered from nephropathy, neuropathy, retinopathy, and CAD either alone or in combination with others. In Alshahrani et al.'s study, 27% of the study population were nephropathic cases [11]. A recent cross-sectional study has highlighted a positive correlation between peripheral neuropathy and poor QoL scores in diabetic mellitus patients [18]. Hypertension, unstable angina, CAD, and myocardial infarction are severe hyperglycemic complications. Hence, final diagnosis and lifestyle changes negatively impact the EQ-5D-5L utility score [10,11].

From the present study cohort, we found comparatively lower 5D-5L scores in the range of 4-10 for 72 (60.5%) participants. Likewise, we got EQ-VAS scores of 70-100 for 72 (60.5%) participants. Though the participants perceived their overall health to be good according to the EQ-VAS scores, the contributory factor may be perceived to be the absence of comorbidities in 64% of the subjects.

Diabetic patients in Muhimbili Medical Centre in Tanzania reported statistically significant poorer health than non-diabetics in eight health domains [19]. In a systematic review of HRQoL by Hernández-Segura et al. in 2022, mobility was noted to be the most important domain among mobility, self-care, and domestic life [6] in diabetic patients. Also, patients with diabetic foot ulcers fared poorly in HRQoL scores than those without, in a study from Sudan [12]. Type 2 diabetes mellitus patients had lower HRQoL owing to old age of 64 years, longer duration of the disease, poor glycemic control, obesity, insulin use, and other diabetes-related complications [13]. In the Saudi Arabian population of Khamis Mushit, type 2 diabetes patients with low QoL had disease duration of more than 10 years, had poor glycaemic control, and bore complication-related organ dysfunctions [11]. In another study in Singapore, pain, discomfort, and diet restrictions were the prevalent complaints among 28% of all the respondents in the five EQ-5D domains [20]. The EQ-VAS scores of normal, prediabetic, and diabetic respondents of a Chinese population were 73.76, 77.45, and 68.34, respectively, with females having higher HRQoL scores than males [17].

A study by Natrajan and Zwane showed that 90% of South Indian type 2 diabetes patients perceived poor HRQoL [21]. Of their patients, 52% and 27% reported diabetes-related distress and discrimination, thereby reflecting upon the importance of patient-centric care from the family to improve their HRQoL. The clinical association was linked to poor glycemic control measures, low energy, mobility, high anxiety, worry, and micro- and macro-vascular complications. Age higher than 50 years, female gender, diabetic foot, and mental depression appeared to be the operators for poor QoL scores in a study by Meher et al., in eastern India [10]. Similarly, in our study located in another part of eastern India, a high glycemic index above 6.5% and an age of more than 45 years correlated with poorer QoL scores. In our study, though, self-perception was satisfactory due to a lack of comorbidities in 64% of the subjects.

Since this is a single-center study, confined to IGIMS, Patna, the characteristic lifestyle and food habits of the particular area might have influenced our results, which may be considered as a limitation. Also, the present cross-sectional study design is limited by its ability to draw any relationship between the cause and the evolving diabetes dynamics. The inclusion of the effects of diet, exercise, and sleep would have added depth to our study.

## Conclusions

The perception of problems in mobility, self-care, usual activities, pain or discomfort, and anxiety or depression is similar in diabetic patients, irrespective of their gender. Good mobility, self-care, and performing usual activities reduce anxiety or depression in contrast to age, pain, and discomfort. In the present study, health statistics derived from the patients in terms of the EQ-VAS scores and 5D-5L scores were overall good despite undergoing medication and some of them suffering from comorbidities. Hence, it can be deduced that they were under better care and diabetes management and that the glycemic control strategies were also working positively for them. Such a questionnaire-related exercise may be mandated as a part of diabetic care toward a more efficient caregiving objective to tackle diabetes.

## Figures and Tables

**Figure 9:**
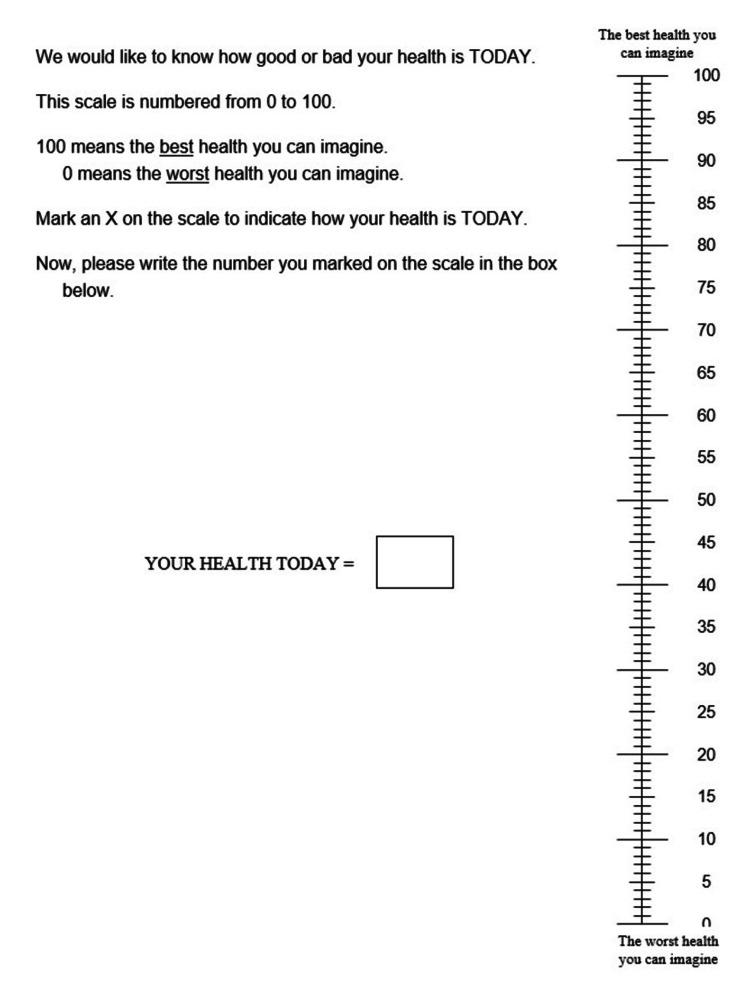
Questionnaire section II (EQ-VAS) EQ-VAS: EuroQol-Visual Analogue Scale

## Appendices

**Table 2:** Questionnaire section I (EQ-5D-5L) EQ-5D-5L: EuroQol 5-dimensions 5-levels

Under each heading, please tick the option that best describes your health today:	
MOBILITY	
I have no problems in walking about	
I have slight problems in walking about	
I have moderate problems in walking about	
I have severe problems in walking about	
I am unable to walk about	
SELF-CARE	
I have no problems in bathing or dressing myself	
I have slight problems in bathing or dressing myself	
I have moderate problems in bathing or dressing myself	
I have severe problems in bathing or dressing myself	
I am unable to bathe or dress myself	
USUAL ACTIVITIES (e.g. work, study, housework, family or leisure activities)	
I have no problems doing my usual activities	
I have slight problems doing my usual activities	
I have moderate problems doing my usual activities	
I have severe problems doing my usual activities	
I am unable to do my usual activities	
PAIN/DISCOMFORT	
I have no pain or discomfort	
I have slight pain or discomfort	
I have moderate pain or discomfort	
I have severe pain or discomfort	
I have extreme pain or discomfort	
ANXIETY/DEPRESSION	
I am not anxious or depressed	
I am slightly anxious or depressed	
I am moderately anxious or depressed	
I am severely anxious or depressed	
I am extremely anxious or depressed	

## References

[1] Williams R, Karuranga S, Malanda B (2023). World Health Organization: Diabetes. Diabetes Res Clin Pract.

[2] Lule SA, Kushitor SB, Grijalva-Eternod CS (2024). The contextual awareness, response and evaluation (CARE) diabetes project: study design for a quantitative survey of diabetes prevalence and non-communicable disease risk in Ga Mashie, Accra, Ghana. Glob Health Action.

[3] Antar SA, Ashour NA, Sharaky M (2023). Diabetes mellitus: classification, mediators, and complications; a gate to identify potential targets for the development of new effective treatments. Biomed Pharmacother.

[4] Gupta J, Kapoor D, Sood V (2021). Quality of life and its determinants in patients with diabetes mellitus from two health institutions of Sub-Himalayan Region of India. Indian J Endocrinol Metab.

[5] (2023). Centre for Disease Control and Prevention, CDC. https://www.cdc.gov/index.htm.

[6] Hernández-Segura N, Marcos-Delgado A, Pinto-Carral A, Fernández-Villa T, Molina AJ (2022). Health-related quality of life (HRQOL) instruments and mobility: a systematic review. Int J Environ Res Public Health.

[7] Kalra S, Jena BN, Yeravdekar R (2018). Emotional and psychological needs of people with diabetes. Indian J Endocrinol Metab.

[8] (2024). EuroQol Research Foundation: EQ-5D user guide. EQ-5D-5L User Guide: Basic Information on How to Use the EQ-5D-5L Instrument, Version 3.0 2019.

[9] Shah B, Deshpande S (2014). Assessment of effect of diabetes on health-related quality of life in patients with coronary artery disease using the EQ-5D questionnaire. Value Health Reg Issues.

[10] Meher D, Kar S, Pathak M, Singh S (2020). Quality of life assessment in diabetic patients using a validated tool in a patient population visiting a tertiary care center in Bhubaneswar, Odisha, India. ScientificWorldJournal.

[11] Alshahrani JA, Alshahrani AS, Alshahrani AM, Alshalaan AM, Alhumam MN, Alshahrani NZ 4th (2023). The impact of diabetes mellitus duration and complications on health-related quality of life among type 2 diabetic patients in Khamis Mushit City, Saudi Arabia. Cureus.

[12] Manjunath K, Christopher P, Gopichandran V, Rakesh PS, George K, Prasad JH (2014). Quality of life of a patient with type 2 diabetes: a cross-sectional study in rural South India. J Family Med Prim Care.

[13] Weinberg Sibony R, Segev O, Dor S, Raz I (2023). Drug therapies for diabetes. Int J Mol Sci.

[14] Perveen W, Ahsan H, Rameen Shahzad (2024). Prevalence of peripheral neuropathy, amputation, and quality of life in patients with diabetes mellitus. Sci Rep.

[15] Bin Rakhis SA Sr, AlDuwayhis NM, Aleid N, AlBarrak AN, Aloraini AA (2022). Glycemic control for type 2 diabetes mellitus patients: a systematic review. Cureus.

[16] Msoka A, Lugina H, Smide B (2006). Assessment of health-related quality of life in people with diabetes and people without diabetes in Tanzania. Int Diabetes Nurs.

[17] Hamid YH, Mohammed M, Hamid S, Mohamedahmed W, Ahmed O (2024). Impact of diabetic foot ulcer on the health-related quality of life of diabetic patients in Khartoum State. Cureus.

[18] Gebremariam GT, Biratu S, Alemayehu M, Welie AG, Beyene K, Sander B, Gebretekle GB (2022). Health-related quality of life of patients with type 2 diabetes mellitus at a tertiary care hospital in Ethiopia. PLoS One.

[19] Shim YT, Lee J, Toh MP, Tang WE, Ko Y (2012). Health-related quality of life and glycaemic control in patients with type 2 diabetes mellitus in Singapore. Diabet Med.

[20] Long E, Feng S, Zhou L (2021). Assessment of health-related quality of life using EuroQoL-5 dimension in populations with prediabetes, diabetes, and normal glycemic levels in Southwest China. Front Public Health.

[21] Natarajan J, Mokoboto-Zwane S (2022). Health-related quality of life and domain-specific associated factors among patients with type2 diabetes mellitus in South India. Rev Diabet Stud.

